# Supplementary material for: By the numbers and in their own words: A mixed methods study of unmet needs and humanitarian inclusion of older Syrian refugees in Lebanon
Source: PLoS One. 2024 Jul 15;19(7):e0302082. doi: 10.1371/journal.pone.0302082 (PMC11249227; doi:10.1371/journal.pone.0302082)
Supplement: S1 Checklist — (DOCX) [file pone.0302082.s001.docx]

Inclusivity in global research

PLOS’ policy on inclusivity in global research aims to improve transparency in the reporting of research performed outside of researchers’ own country or community and ensures that PLOS publications reporting global research adhere to high standards for research ethics and authorship. Authors of relevant research articles may be asked to complete the questionnaire below, which outlines ethical, cultural, and scientific considerations specific to inclusivity in global research. This questionnaire may be requested when researchers have travelled to a different country to conduct research, if research uses samples collected in another country, research with Indigenous populations or their lands, or if research is on cultural artefacts. Researchers travelling to another country solely to use laboratory equipment will not normally be required to complete the questionnaire. However, the questionnaire can be requested at the journal’s discretion for any submission – if you have been requested to complete this questionnaire by the PLOS journal you submitted to, please do so.

Please complete the questionnaire below and include this as a Supporting Information file with your manuscript. Note that if your paper is accepted for publication, this checklist will be published with your article in the supporting information files. Please ensure that you reference the checklist in the main body of your manuscript. We suggest adding a subsection ‘Inclusivity in global research’ to your Methods section and adding the following sentence: “Additional information regarding the ethical, cultural, and scientific considerations specific to inclusivity in global research is included in the Supporting Information (SX Checklist)”

The questions have been designed to be applicable to a wide range of study types, and there are subsections for both human subjects research and non-human subjects research. If any of the questions are not relevant to your research please mark them as “N/A” as appropriate.

**Ethical considerations, permits and authorship**

*This section is applicable to all research types.*

Provide details as to who granted permissions and/or consent for the study to take place in the Methods section of your manuscript. This should include the names of **all** ethics boards, governmental organizations, community leaders or other bodies that provided approval for the study. If individuals provided approval refer to these people by their role or title but do not list their name(s).

Reported on page number: 12 (lines 242-243)

If there were any deviations from the study protocol after approval was obtained please provide details of these changes in the Methods section of your manuscript.
Did this study involve local collaborators that are residents of the country where the research was conducted or members of the community studied? If you do not have any authors from said communities, please provide an explanation for this below.

Not applicable as there were no deviations from the approved study protocol.

Yes – All co-authors are in Lebanon.

Everyone listed as an author should meet PLOS’ criteria for authorship and all individuals who meet these criteria should be included in the author byline, rather than the acknowledgements. Authorship criteria is based on the International Committee of Medical Journal Editors (ICMJE) Uniform Requirements for Manuscripts Submitted to Biomedical Journals - for further information please see here: <https://journals.plos.org/plosone/s/authorship>.

**Human subjects research (e.g. health research, medical research, cross-cultural psychology)**

Did you obtain written informed consent from a representative of the local community or region before the research took place? How did you establish who speaks for the community? Details of written informed consent obtained from study participants should be reported separately in the Methods section of your manuscript.

In line with the proposal approved by the Social and Behavioral Institutional Review Board at the American University of Beirut (ID: SBS-2021-0214), we obtained verbal consent from all research participants, due to COVID-19 consderations at the time and the vulnerability of the older refugee population. These details are reported on page 12 (lines 253-257) in the Methods section. The local communities were all informed and sensitized to the aims of the research beforehand by coordinating with the chiefs of informal tented settlements and hosted communities.

How did members of the local community provide input on the aims of the research investigation, its methodology, and its anticipated outcome(s)?

Feedback on the research methodology and approach was solicited from the local humanitarian agency we have coordinated with for access to the communities. This agency has a longstanding experience servicng the refugee communities. Second, once tools were developed, they were pilot tested with humanitarian field workers who have worked with the local community, and with local community members (both male and female), and they also provided inputs on the tool.

When engaging with the local community, how did you ensure that the informed consent documents and other materials could be understood by local stakeholders?

We worked sensitively with community members taking part in the research to take time to explain the study to them. The entire research team as well as the data collectors are native Arabic speakers and speak Levantine Arabic which is the dialect spoken in both Lebanon and Syria. The data collectors were also trained on how to explain extensively but in simple terms the informed consent. After the explanation, three questions on the consent were asked to each interviewee to makes usre they understood their rights in participating in the study. Only when the three questions were answered correctly did the interview begin.

Will the findings of the research be made available in an understandable format to stakeholders in the community where the study was conducted (e.g. via a presentation, summary report, copies of publications, etc.)? Please provide details of how this will be achieved.

Since it is hard to track back the participants, we did not plan to disseminate the findings among the participants. However, we will be sharing the results of our study in the form of key messages as well as the publications with the NGO that has supported our data collection activities and which provides direct services to the community. The findings would be relevant to their practice and interventions.

**Non-human subjects research using specimens/ animals collected as part of the study, or those housed in archival collections. Examples include archaeology, paleontology, botany and zoology.**

Did the permission you obtained from a local authority to perform the study include an agreement on access to outputs and benefit sharing? This may include procedures to enable fair distribution of the benefits and resources arising from the research performed. Please include any details of Prior Informed Consent and Benefit Sharing Agreements obtained. These may be required by field-specific regulations, for example the Convention on Biological Diversity (CBD) and the associated Nagoya Protocol.

N/A

If the material used in your study was imported, please A) provide the year it was imported and B) indicate whether permits were obtained to import/export the materials used, C) provide details of any permits obtained. If this information is not available, please indicate this.

N/A

If you used archival specimens, please state how the material used in your study was acquired by the institute it is held in and provide details of any permits obtained for the original excavations/ sample collection. If this information is not available, please indicate this.

N/A

How was the potential cultural significance of the materials collected in your study to local communities considered in your research design? Were Indigenous peoples and/or local researchers and institutions involved with archaeological excavations / collection of specimens? If so, please provide a description of their involvement.

N/A

If your manuscript includes photographs of human remains please indicate whether authors obtained permission from descendants or affiliated cultural communities to do so.

N/A
