# Supplementary material for: By the numbers and in their own words: A mixed methods study of unmet needs and humanitarian inclusion of older Syrian refugees in Lebanon
Source: PLoS One. 2024 Jul 15;19(7):e0302082. doi: 10.1371/journal.pone.0302082 (PMC11249227; doi:10.1371/journal.pone.0302082)
Supplement: S2 Checklist — (DOCX) [file pone.0302082.s002.docx]

| **Topic** | **Item no.** | **Guide questions/ Description** | **Notes** | **Reported on page No.** |
| --- | --- | --- | --- | --- |
| **Domain 1: Research team and reflexivity** | | | |  |
| *Personal characteristics* | | | |  |
| Interviewer/facilitator | 1 | Which author/s conducted the interview or focus group? | The first author, SAO, conducted the interviews. | 11 |
| Credentials | 2 | What were the researcher’s credentials? E.g. PhD, MD | The researcher (SAO) was a PhD candidate in Epidemiology at the American University of Beirut. | 11 |
| Occupation | 3 | What was their occupation at the time of the study? | The researcher (SAO) was a PhD candidate in Epidemiology at the American University of Beirut. | 11 |
| Gender | 4 | Was the researcher male or female? | SAO is female. | 11 |
| Experience and training | 5 | What experience or training did the researcher have? | SAO was already trained and experienced in conducting qualitative interviews and data collection including key informant interviews and facilitating focus group discussions. | 11 |
| *Relationship with participants* | | | |  |
| Relationship established | 6 | Was a relationship established prior to study commencement? | No. No relationship was established before study commencement. | 11 |
| Participant knowledge of the interviewer | 7 | What did the participants know about the researcher? e.g. personal goals, reasons for doing the research | The participants were informed that the researcher is conducting this study to understand the needs of OSRs and their experiences and inclusion in the humanitarian response in Lebanon. | 11 |
| Interviewer characteristics | 8 | What characteristics were reported about the inter viewer/facilitator? e.g. Bias, assumptions, reasons and interests in the research topic | The participants were informed that the researcher is conducting this study to understand the needs of OSRs and their experiences and inclusion in the humanitarian response in Lebanon. | 11 |
| **Domain 2: Study design** | | | |  |
| *Theoretical framework* | | | |  |
| Methodological orientation and Theory | 9 | What methodological orientation was stated to underpin the study? e.g.  grounded theory, discourse analysis, ethnography, phenomenology,  content analysis | A descriptive methodology was used in this study. | 10 |
| *Participant selection* | | | |  |
| Sampling | 10 | How were participants selected? e.g. purposive, convenience, consecutive, snowball | Participants were selected purposefully among those who participated in the quantitative study such that: they are informative individuals, from both genders, from both Northern areas and Bekaa, and they cover a wide age range (50 and above). | 10 |
| Method of approach | 11 | How were participants approached? e.g. face-to-face, telephone, mail, email | Participants were approached face-to-face in their households. | 11 |
| Sample size | 12 | How many participants were in the study? | A total of 14 participants were included in the study. | 11 |
| Non-participation | 13 | How many people refused to participate or dropped out? Reasons? | None of the approached OSRs refused to participate. | 11 |
| *Setting* | | | |  |
| Setting of data collection | 14 | Where was the data collected? e.g. home, clinic, workplace | Data were collected at participants’ households | 11 |
| Presence of non-participants | 15 | Was anyone else present besides the participants and researchers? | No. No one was present besides the participants and researchers during the interviews. | 11 |
| Description of sample | 16 | What are the important characteristics of the sample? e.g. demographic data, date | The participants’ ages ranged between 53 and 72. ## were female. ## resided in ITSs, and ## resided in Bekaa. | Table 2 on pages 17-18 |
| Data collection | | | |  |
| Interview guide | 17 | Were questions, prompts, guides provided by the authors? Was it pilot tested? | The topic guide was conceptualized and designed by SAO. It was not pilot-tested, but it was pre-tested with a group of three humanitarian field workers who have experience working with OSRs in Lebanon. | 11 |
| Repeat interviews | 18 | Were repeat interviews carried out? If yes, how many? | No repeat interviews were conducted. | N/A |
| Audio/visual recording | 19 | Did the research use audio or visual recording to collect the data? | The research used audio-recording of the interviews. | 11 |
| Field notes | 20 | Were field notes made during and/or after the interview or focus group? | Field notes were made during the interviews. | 11 |
| Duration | 21 | What was the duration of the interviews or focus group? | An interview took, on average, 30-40 minutes. | 11 |
| Data saturation | 22 | Was data saturation discussed? | Data saturation was reached on all the major topics. | 11 |
| Transcripts returned | 23 | Were transcripts returned to participants for comment and/or correction? | No. The transcripts were not returned to the participants for comment and/or correction. | N/A |
| **Domain 3: analysis and findings** | | | |  |
| *Data analysis* | | | |  |
| Number of data coders | 24 | How many data coders coded the data? | SAO coded the data. | 12 |
| Description of the coding tree | 25 | Did authors provide a description of the coding tree? | Yes | Provided as supplementary material S3 |
| Derivation of themes | 26 | Were themes identified in advance or derived from the data? | A deductive approach was used where main themes were identified in advance. | 11-12 |
| Software | 27 | What software, if applicable, was used to manage the data? | None. | N/A |
| Participant checking | 28 | Did participants provide feedback on the findings? | No, participants did not provide feedback on the findings. | N/A |
| Reporting | | | |  |
| Quotations presented | 29 | Were participant quotations presented to illustrate the themes/findings? Was each quotation identified? e.g. participant number | Yes. Key findings were supported with selected quotes in text. | Across all the results sub-sections (p. 20-43) |
| Data and findings consistent | 30 | Was there consistency between the data presented and the findings? | Yes. All findings were derived from the data and supported by illustrative quotes. | Across all the results sub-sections (p. 20-43) |
| Clarity of major themes | 31 | Were major themes clearly presented in the findings? | Yes. Major themes were derived from the data and clearly defined by corresponding section/paragraph titles. | Across all the results sub-sections (p. 20-43) |
| Clarity of minor themes | 32 | Is there a description of diverse cases or discussion of minor themes? | Yes. Sub-themes that were described by only one participant were also described in the results section of this study. | 24-25-43 |
