# Supplementary material for: By the numbers and in their own words: A mixed methods study of unmet needs and humanitarian inclusion of older Syrian refugees in Lebanon
Source: PLoS One. 2024 Jul 15;19(7):e0302082. doi: 10.1371/journal.pone.0302082 (PMC11249227; doi:10.1371/journal.pone.0302082)
Supplement: S3 Checklist — (DOCX) [file pone.0302082.s003.docx]

**Appendix S1. Good Reporting of A Mixed Methods Study (GRAMMS) checklist**

| **Guideline** | **Section: page** |
| --- | --- |
| Describe the justification for using a mixed methods approach to the research question | Introduction: p. 5 |
| Describe the design in terms of the purpose, priority and sequence of methods | Methods: p. 6 |
| Describe each method in terms of sampling, data collection and analysis | Methods: p. 7-13 |
| Describe where integration has occurred, how it has occurred and who has participated in it | Methods: p. 12 |
| Describe any limitation of one method associated with the present of the other method | Discussion: p. 49 |
| Describe any insights gained from mixing or integrating methods | Discussion: p. 44-48 |

O'Cathain A, Murphy E, Nicholl J. The quality of mixed methods studies in health services research. J Health Serv Res Policy. 2008;13: 92-98
