# Supplementary material for: By the numbers and in their own words: A mixed methods study of unmet needs and humanitarian inclusion of older Syrian refugees in Lebanon
Source: PLoS One. 2024 Jul 15;19(7):e0302082. doi: 10.1371/journal.pone.0302082 (PMC11249227; doi:10.1371/journal.pone.0302082)
Supplement: S1 Appendix — (DOCX) [file pone.0302082.s004.docx]

**Narrative description of the coding tree pertaining to the qualitative findings**


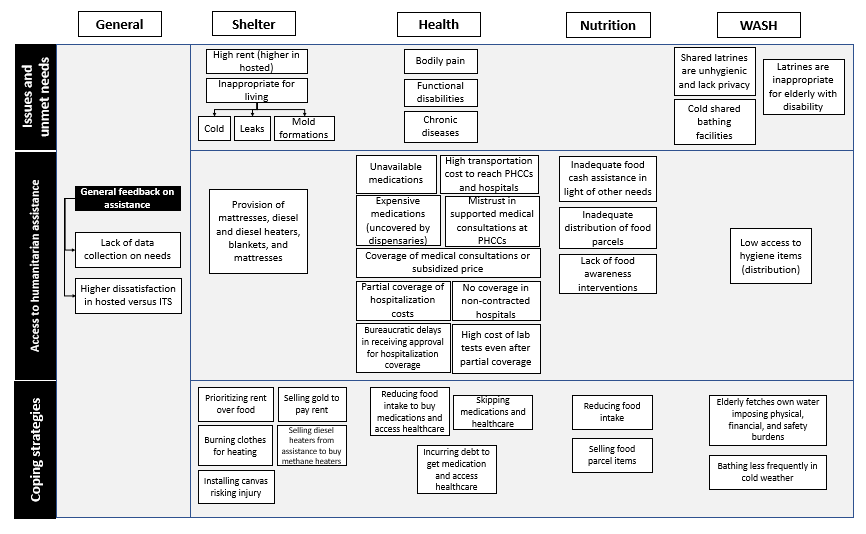


The coding tree presents a detailed categorization of issues and unmet needs concerning humanitarian assistance for older Syrian refugees in Lebanon. The tree is structured into five primary categories: General, Shelter, Health, Nutrition, and WASH (Water, Sanitation, and Hygiene). Each category further breaks down into specific issues, feedback on assistance, and coping strategies.

**1. General**

- **Issues and Unmet Needs**: General feedback on assistance includes:
  - Lack of data collection on needs.
  - Higher dissatisfaction in hosted environments compared to Informal Tented Settlements (ITS).

**2. Shelter**

- **Issues and Unmet Needs**:
  - High rent, particularly in hosted settings.
  - Inappropriate living conditions characterized by:
    - Cold
    - Leaks
    - Mold formations
- **Access to Humanitarian Assistance**:
  - Provision of mattresses, diesel and diesel heaters, blankets, and mattresses.
- **Coping Strategies**:
  - Prioritizing rent over food.
  - Selling gold to pay rent.
  - Burning clothes for heating.
  - Selling diesel heaters from assistance to buy methane heaters.
  - Installing canvas despite risking injury.

**3. Health**

- **Issues and Unmet Needs**:
  - Bodily pain.
  - Functional disabilities.
  - Chronic diseases.
- **Access to Humanitarian Assistance**:
  - Unavailable medications.
  - Expensive medications not covered by dispensaries.
  - Partial coverage of hospitalization costs.
  - Bureaucratic delays in receiving approval for hospitalization coverage.
  - High transportation costs to reach Primary Health Care Centers (PHCCs) and hospitals.
  - Mistrust in supported medical consultations at PHCCs.
  - No coverage in non-contracted hospitals.
  - High cost of lab tests even after partial coverage.
- **Coping Strategies**:
  - Reducing food intake to buy medications and access healthcare.
  - Skipping medications and healthcare.
  - Incurring debt to get medication and access healthcare.

**4. Nutrition**

- **Issues and Unmet Needs**:
  - Inadequate food cash assistance considering other needs.
  - Inadequate distribution of food parcels.
  - Lack of food awareness interventions.
- **Coping Strategies**:
  - Reducing food intake.
  - Selling food parcel items.

**5. WASH (Water, Sanitation, and Hygiene)**

- **Issues and Unmet Needs**:
  - Shared latrines are unhygienic and lack privacy.
  - Latrines are inappropriate for elderly individuals with disabilities.
  - Cold shared bathing facilities.
  - Low access to hygiene items.
- **Coping Strategies**:
  - Elderly fetching their own water, which imposes physical, financial, and safety burdens.
  - Bathing less frequently in cold weather.

This coding tree effectively highlights the complex and interrelated issues faced by older Syrian refugees in Lebanon, detailing their unmet needs, the limitations of current humanitarian assistance, and the various coping strategies employed to manage their difficult circumstances.
